# Dataset for the establishment of an age model of marine sediment core KH19-6 Leg.4 PC10/MC14 collected from the Agulhas Ridge in the South Atlantic Ocean

**DOI:** 10.1016/j.dib.2022.108797

**Published:** 2022-12-05

**Authors:** Kaoru Kubota, Rosaaideihn Tanabe, Minoru Ikehara, Yukiko Kozaka, Koji Seike, Yosuke Miyairi, Yusuke Yokoyama

**Affiliations:** a Japan Agency for Marine-Earth Science and Technology, 2-15 Natsushima, Yokosuka, Kanagawa 237-0061, Japan; b Graduate School of Human Development and Environment, Kobe University 3-11 Tsurukabuto, Nada, Kobe, Hyogo 657-8501, Japan; c Center for Advanced Marine Core Research, Kochi University, 200 Monobu-otsu, Nankoku Kochi 783-8502, Japan; d National Institute of Advanced Industrial Science and Technology (AIST), AIST Tsukuba Central 7, 1-1-1 Higashi, Tsukuba 305-8567, Japan; e Atmosphere and Ocean Research Institute, The University of Tokyo, 5-1-5 Kashiwa, Chiba 277-8564, Japan

**Keywords:** Radiocarbon, Oxygen isotope, Marine sediment core, Agulhas Ridge, Foraminifera, *Gyroidina soldanii*, *Globigerinoides bulloides*, *Globorotalia inflata*

## Abstract

A precise age model of marine sediment core is crucial for environmental studies of the past such as paleoceanography, paleoclimatology, and paleo-hazard studies. Here the geochemical dataset is described that is used to determine the age model of marine sediment cores collected from Agulhas Ridge in the South Atlantic Ocean using piston coring and multiple-coring systems during the 30th Anniversary expeditions of R/V Hakuho Maru in 2019–2020 (KH19-6 Leg.4 PC10/MC14, water depth of 4,604 m). The top 3.27 meter of 12.28-meter-long piston core (PC10) and a whole 0.29-m-long multiple core (MC14) were dated. The dataset includes radiocarbon ages of planktonic foraminifera shells and oxygen isotopes of both planktonic (*Globigerinoides bulloides, Globorotalia inflata*) and benthic (*Gyroidina soldanii*) foraminifera shells. The top 7.5 kyr record was lost, the ages of 3.27 m depth below sea floor was ∼140 kyr ago, and sedimentation rates were 0.9–5.5 kyr/cm.

**Specifications Table**

**Table:** 

Subject	Earth and Planetary Sciences > Stratigraphy
Specific subject area	Stratigraphy of marine sediment core using geochemical approach including radiocarbon dating.
Type of data	Figure
How the data were acquired	*The marine sediment core samples were collected during an expedition of R/V Hakuho maru (KH19-6 Leg.4). After getting off the ship, the sediment samples were cut into subsamples and fossil foraminifera shells were collected in the laboratory. By hand-picking target species’ shells, these were dissolved in phosphoric acid and the emitted carbon dioxide were used for both radiocarbon and stable oxygen (and carbon) isotopic analysis. Radiocarbon and stable oxygen isotopes were measured by using accelerator mass spectrometry (AMS) and isotope ratio mass spectrometry (IR-MS), respectively.* *A cruise report of KH19-6 Leg.4 is available at*https://www.godac.jamstec.go.jp/cr_catalog/view/metadata?key=KH-19-6_leg4_all&lang=en
Data format	*Raw* *Analyzed*
Description of data collection	*Total 24 radiocarbon analyses for planktonic foraminifera from a multiple corer as well as a piston corer. One radiocarbon date was regarded as outlier it was out of stratigraphic consistency, likely due to a disturbance during the piston coring.* *Total 60 oxygen (and carbon) isotope analyses for planktonic foraminifera, Globigerinoides bulloides, of PC10.* *Total 169, oxygen (and carbon) isotope analyses for planktonic foraminifera, Globorotalia inflata, of PC10 and MC14.* *Total 169, oxygen (and carbon) isotope analyses for planktonic foraminifera, Gyroidina soldanii, of PC10 and MC14.* *Total 104 data points of weight percentage of the coarse fraction (>63 µm) of PC10.*
Data source location	*Marine sediment cores (PC10 and MC14)* • *Collection Date: 14 Jan 2020* • *Latitude: 39°38.2465’S (-39.6374)* • *Longitude: 14°28.4717’E (14.4822)* • *Water depth: 4,604 m* *Radiocarbon analyses* • *Institution: Atmosphere and Ocean Research Institute, The University of Tokyo* • *City: Kashiwa, Chiba* • *Country: Japan* *Oxygen and carbon isotope analyses, coarse fraction of PC10* • *Institution: Center for Advanced Marine Core Research, Kochi University* • *City: Nankoku, Kochi* • *Country: Japan*
Data accessibility	*Repository name: PANGAEA**[1]* *Data identification number:*10.1594/PANGAEA.949117 *Direct URL to data:*https://doi.org/10.1594/PANGAEA.949117

## Value of the Data

• The age model of marine sediment cores is a prerequisite for relevant studies using this material.
• Paleoenvironmental records based on a robust age model will contribute to an accurate understanding of environmental changes of the past.
• These data are useful to those who plan to collect new sediment cores from South Atlantic site in the future.

## 1 Objective

An age model of marine sediment core is a prerequisite to start environmental studies of the past such as paleoceanography, paleoclimatology, and paleo-hazard studies. It helps to understand chronological sequence of an event that happened in the past. Also, it reveals sedimentation rate of the cores, which helps to determine time intervals to study and how much sediment materials need to be collected during sampling to achieve the desired temporal resolution.

## 2 Data Description

All geochemical data obtained are archived in an online repository, which include radiocarbon dates of planktonic foraminifera, oxygen and carbon isotopes of both planktonic and benthic foraminifera, and coarse fraction of the sediment.

## 3 Experimental Design, Materials and Methods

### 3.1 Materials

Marine sediment cores (PC10 and MC14) obtained from the Agulhas Ridge in 14 Jan 2020 by R/V Hakuho maru were analyzed.

**PC10:** A piston corer consists of a 900 kg-weight, a total 14 m-long stainless steel barrel and polyvinyl chloride (PVC) inner pipe, a core bit, a core catcher, a wired piston cylinder inside and a trigger arm. The inner diameter of inner pipe is 74 mm. A 100-kg weighed multiple-type corer “Asyura” was used as pilot weight. The multiple-type pilot corer consists of a stainless-steel frame and three sub-corer attachments containing three 0.6-m long acrylic resin tubes and self-closing lids at both ends of each tube, which enables a collection of short sediment cores and bottom water without disturbance at the sediment surface. The inner diameter of each acrylic resin tube is 80 mm. A 12.28-m-long PC10 was obtained, and the core was split into working- and archive-halves using a splitting devise with nylon line. Archive-half sections was provided for photographs, color reflectance measurements with a reflectance photospectrometer and visual core description of lithology (clay with foraminifera diatoms). Working-half sections was used for a further sampling: The sediment was cut in 1 cm intervals and used for further analyses.

**MC14:** Surface sediment samples were collected with a Barnet-type multiple corer to minimize a sediment disturbance during the corer penetration into sediments. The multiple corer equipped eight 60-cm long core tubes with an inner diameter of 82 mm. A 74 mm inner diameter tube was used. After recovery of the multiple corer onboard, we retrieved sediments with overlying seawater and kept chilled either outside the deck or in a cold room. 0.29-meter-long MC14 was obtained. A whole MC14 were used for further analyses: The sediment was cut in 1 cm intervals and used for further analyses.

### 3.2 Coarse Fraction Analysis of PC10

In a laboratory at the Center for Advanced Marine Core Research, Kochi University, the weight of each subsample was measured after drying. They were gently washed under running water over a 63 µm sieve (SIEVE FACTORY IIDA Co., Ltd., Japan) and dried at 60°C in a convection oven (Yamato Scientific Co., Ltd., Japan). The weights of them were measured, and the weight percent of coarse material was calculated.

### 3.3 Radiocarbon Analysis of PC10 and MC14

In a laboratory at Kobe University, the subsamples of PC10 and MC14 were freeze-dried (FDU-2000, EYELA, Japan) and gently washed under running water over a 63 µm sieve (SIEVE FACTORY IIDA Co., Ltd., Japan) and dried at 60°C in a convection oven (Yamato Scientific Co., Ltd., Japan). The sieved materials were further divided into size fractions of 63–255, 255–300, 300–355, 355–425, >425 µm, and stored in plastic vials. From 300–355, 355–425, and >425 µm size fractions, ∼10 mg of planktonic foraminifera, *Globorotalia inflata*, shells were hand-picked by a fine brash under a microscope. Priority were the larger size individuals, as the picking was easier and there is no size-dependency of radiocarbon of foraminiferal tests.

Subsequent radiocarbon analysis was conducted at a laboratory of Atmosphere and Ocean Research Institute (AORI), The University of Tokyo. The shells were gently crushed to open all chambers between grass slides and transferred into vacuum vials. The samples ultrasonicated with Milli-Q water and supernatant with suspended materials was removed. This was repeated several times until the supernatant becomes transparent. After vacuuming air in the vial, the shells were reacted with phosphoric acid, and the carbon dioxide produced was collected. The carbon dioxide was converted to graphite by a Fe catalyst at 620°C for 6–12 hours. The target graphite samples were measured with a single-stage AMS (NEC, USA) installed at AORI.

As for the sample from 25–26 cm depth of MC14, radiocarbon was measured twice (∼10 mg each *G. inflata* shells were consumed), and an average of ^14^C ages was used.

Conventional ^14^C ages were converted into calendar age before 1950 CE using a Marine20 calibration curve [6] with a local marine ^14^C reservoir age of 2 ± 54 years.

### 3.4 Oxygen and Carbon Isotope Analysis of PC10 and MC14

From the coarse fraction of PC10, ∼20 individuals of planktonic foraminifera (*Globigerinoides bulloides* and *Globorotalia inflata*) shells were hand-picked by a fine brash under a microscope. Analysis intervals were 1 cm for both *G. bulloides* and *G. inflata* until ∼50 cm below seafloor, and then 8 cm for *G. bulloides*. and 2–4 cm for *G. inflata. G. bulloides* disappeared deeper than the depth of 124 cm. As for *G. inflata*, intervals equivalent to Marine Isotope Stage 6 to 5 transition were analyzed in higher resolution.

From the coarse fraction of MC14, ∼20 individuals of planktonic foraminifera, *Globorotalia inflata*, shells were hand-picked by a fine brash under a microscope. Analysis intervals were 1 cm.

From the coarse fraction of PC10 and MC14, 3 individuals of benthic foraminifera, *Gyroidina soldanii*, shells were hand-picked by a fine brash under a microscope. Analysis intervals depended on their occurrence. In some samples, they were absent.

The shells were gently crushed to open all chambers between grass slides and transferred into v-shaled vials for oxygen and carbon isotope analyses. Isotopes of planktonic and benthic foraminifera were measured using an isotope-ratio mass spectrometer (Isoprime) with an automated carbonate reaction system (Multiprep) installed at the Center for Advanced Marine Core Research, Kochi University. The values of δ^18^O are reported with respect to the Vienna Pee Dee Belemnite standard using standard delta notation in permil54. Analytical precision for long-term measurement of the calcite standard (IAEA603) was better than 0.1‰. A drift correction for the sample δ^18^O values was performed, as the long-term drift of the measured IAEA603 values of the standards during the measurement period between December 2020 and October 2021 was observed.

### 3.5 Data Screening and Age Model Establishment of PC10 and MC14

**Fig. 1:**
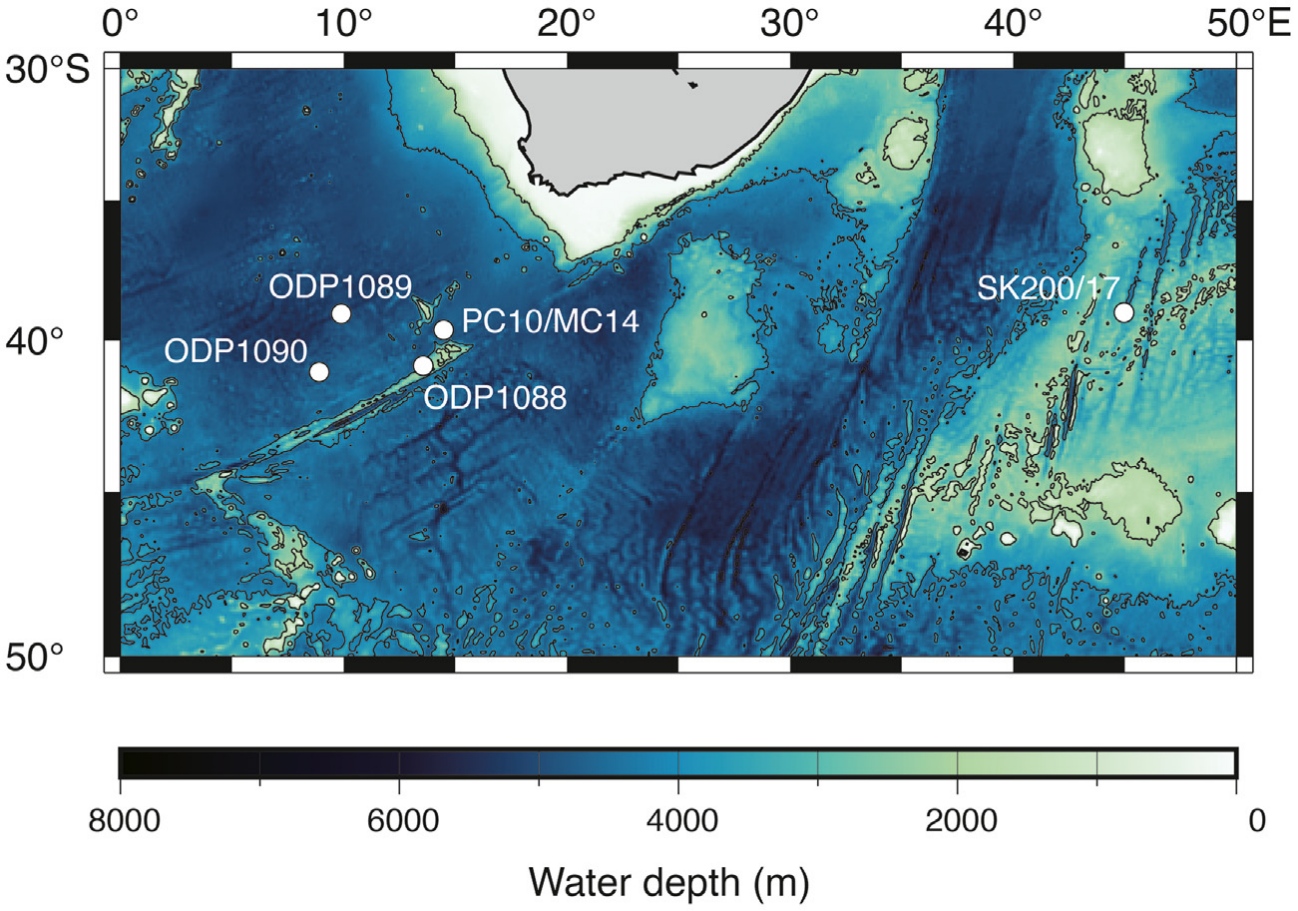
A geographical map of the South Atlantic Ocean and locations of relevant marine sediment cores: PC01 and MC14 (this dataset), ODP1088–1090 [2], and SK200/17 [3].

**Fig. 2:**
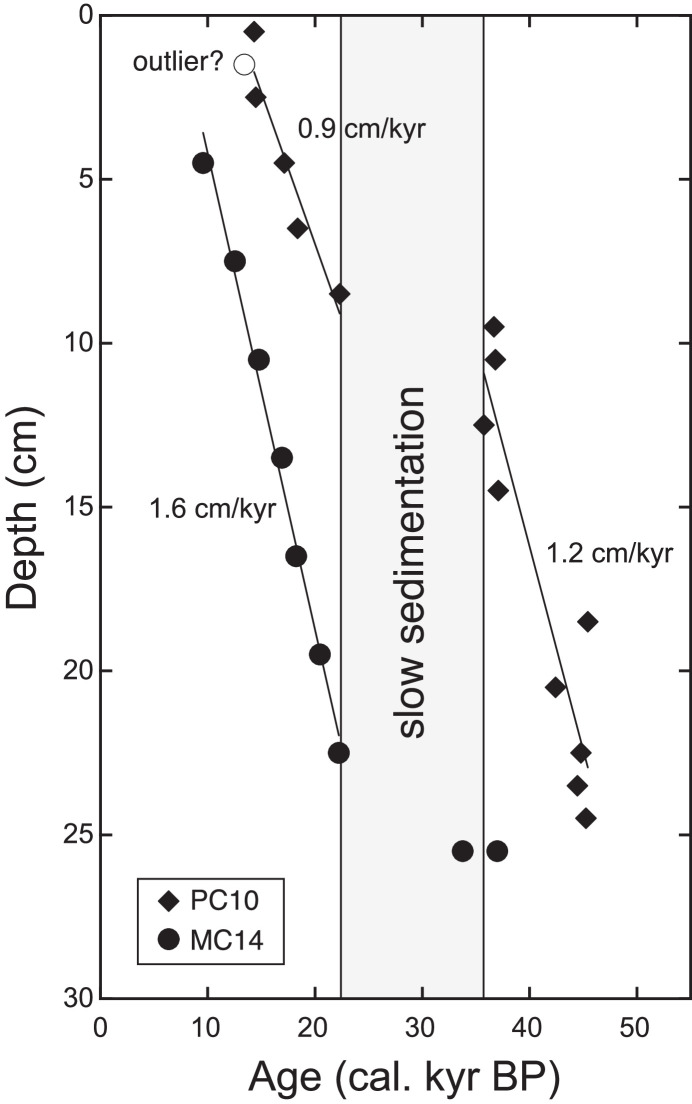
Age-depth models of PC10 (diamonds) and MC14 (circles) based on radiocarbon dating of planktonic foraminifera (*G. inflata*). An outlier at 1–2 cm depth of MC14 was removed from the calculation. A slow sedimentation span was found in at 9 cm depth of PC10 and 22–26 cm depth of MC14, which correspond to the time-period from 21.3 to 35.6 kyr BP.

**Fig. 3:**
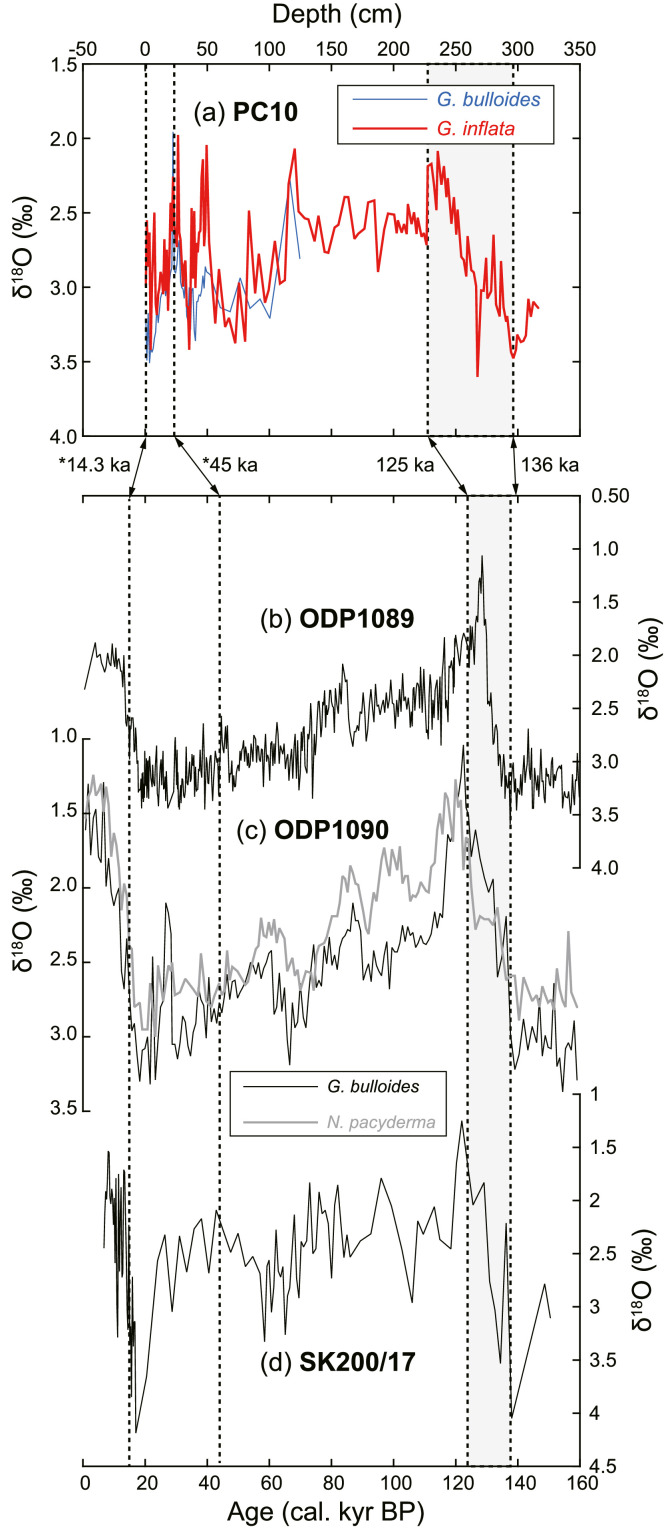
(a) Oxygen isotope variation of *G. bulloides* and *G. inflata* of PC10. Horizontal axis is core depth. (b–d) Oxygen isotope records of planktonic foraminifera of marine sediment cores that were collected from nearby sites (ODP1088–1090 [2]; SK200/17 [3]). By comparing these records, 235–236 cm and 296–297 cm depths of PC10 were tied to 125 kyr BP (Marine Isotope Stage 5, MIS 5) and 136 kyr BP (MIS 6), respectively.

**Fig. 4:**
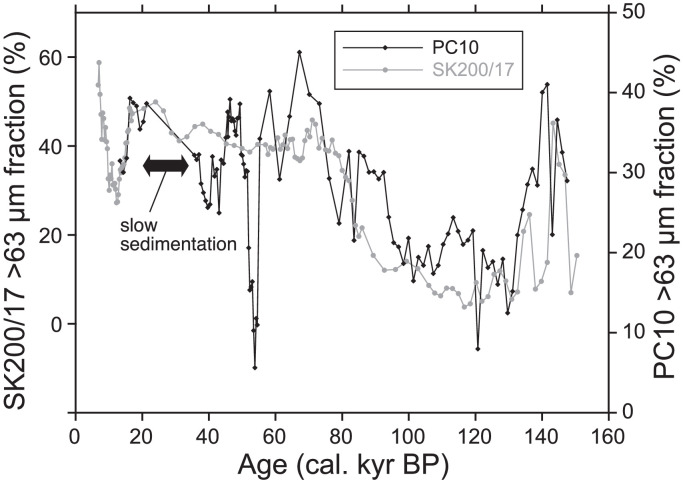
Temporal variation of coarse fraction (> 63 µm) of PC10 in comparison with that of the sediment core SK200/17. A close match of these records supports a robustness of the age model of PC10 Note that the age model of SK200/17 sediment core is based on a comparison between oxygen isotope variation of *G. bulloides* with those of planktonic foraminifera of global low latitude ocean [4], as well as with those of benthic foraminifera of global ocean (LR04 [5]).

**Fig. 5:**
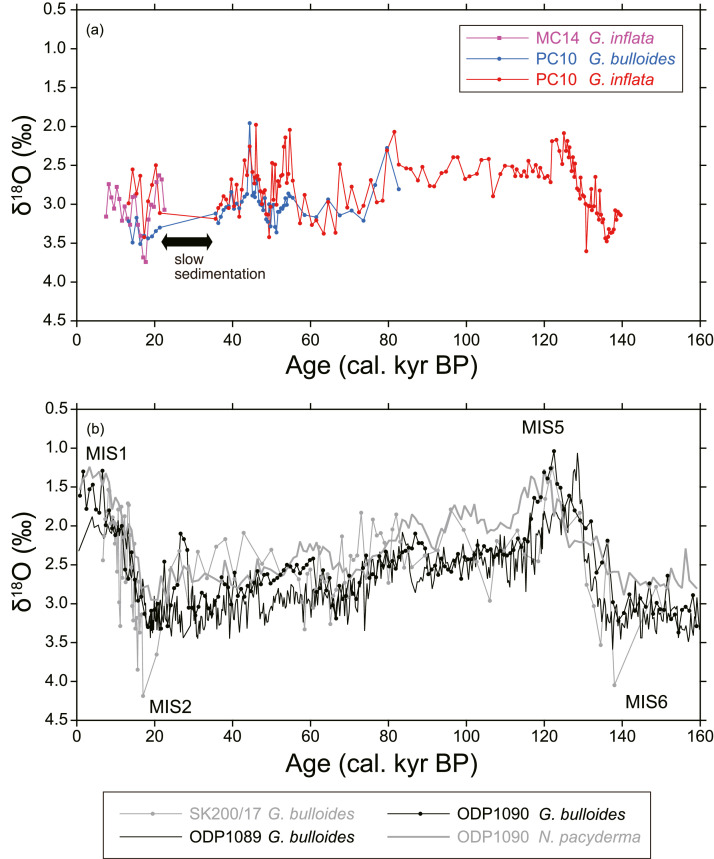
(a) Temporal variation of oxygen isotopes of planktonic foraminiferas *G. bulloides* of PC10 and *G. inflata* of PC10 and MC14. (b) Temporal variation of oxygen isotopes of *G. bulloides* of SK200/17, ODP1089, and ODP1090 and *N. pacyderma* of PC1090. MIS, Marine Isotope Stage.

**Fig. 6:**
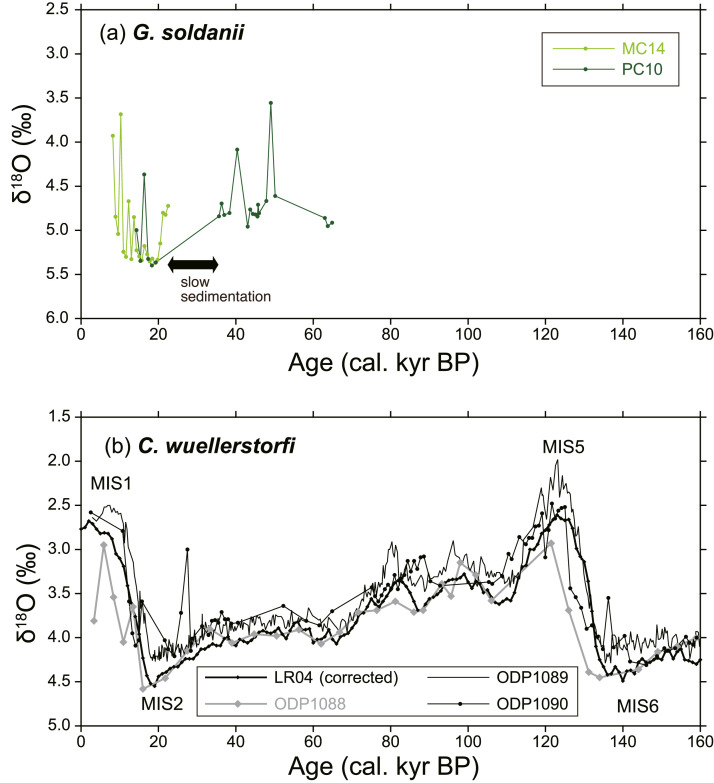
(a) Temporal variation of oxygen isotopes of benthic foraminifera *G. soldanii* of PC10 and MC14. (b) Temporal variation of oxygen isotopes of *Cibicidoide wullestorfi* of ODP1088, ODP1089, and ODP1090. LR04 (for benthic foraminifera *Uvigerina* spp.) was empirically corrected by subtracting 0.5‰ to fit with oxygen isotope curves of *C. wullestorfii*. MIS, Marine Isotope Stage.

As the ^14^C age of 1–2 cm depth of MC14 was out of stratigraphic consistency (older than the samples below), it was omitted from calculations of the age-depth model and sedimentation rate. The age-depth model for MC14 was calculated by linear regression of 8 radiocarbon dates (a sedimentation rate during this span was 1.6 cm/kyr). The age-depth model for PC10 was calculated for two time intervals; one is 0–9 cm depths and the other is 9–25 cm depths (sedimentation rates were 0.9 cm/kyr and 1.2 cm/kyr, respectively). We observed a low sedimentation span at 9 cm depth below seafloor (from 21.3 to 35.6 kyr BP). Below 25 cm depth, 235–236 cm and 296–297 cm depths were tied to 125 kyr BP and 136 kyr BP, respectively. They correspond to marine isotope stage 5 and 6, respectively. The linear sedimentation rates for the intervals 25–236 cm and 236–297 cm were calculated as 2.6 cm/kyr and 5.5 cm/kyr, respectively (Fig. 1, Fig. 2, Fig. 3, Fig. 4, Fig. 5, Fig. 6).

## Ethics Statements

We ensure that we report an entirely original work that has never been published by any media. We declare that the dataset is not relevant to animal subjects, because we don't use any living animals.

## CRediT authorship contribution statement

**Kaoru Kubota:** Conceptualization, Data curation, Methodology, Writing – original draft, Investigation, Visualization, Funding acquisition. **Rosaaideihn Tanabe:** Methodology, Writing – review & editing. **Minoru Ikehara:** Conceptualization, Data curation, Methodology, Validation, Investigation, Resources, Supervision, Writing – review & editing, Project administration, Funding acquisition. **Yukiko Kozaka:** Methodology, Investigation, Writing – review & editing. **Koji Seike:** Data curation, Methodology, Investigation, Writing – review & editing. **Yosuke Miyairi:** Data curation, Methodology, Validation. **Yusuke Yokoyama:** Data curation, Methodology, Validation, Resources, Writing – review & editing, Funding acquisition.

## Declaration of Competing Interest

The authors declare that they have no known competing financial interests or personal relationships that could have appeared to influence the work reported in this paper.

## Data Availability

Dataset used to obtain an age model of marine sediment core KH19-6 Leg 4 PC10/MC14 from the Agulhas Ridge in the South Atlantic Ocean (Original data) (PANGAEA).
